# The relationship between college students’ self-identity and future orientation: a moderated chain-mediating model

**DOI:** 10.3389/fpsyg.2025.1461159

**Published:** 2025-04-04

**Authors:** Peiyu Qiu, Huan Wang, Huarong Wang, Lvqing Miao, Jian Song

**Affiliations:** ^1^Student Affair Office, Nantong University, Nantong, Jiangsu, China; ^2^School of Education Science, Nantong University, Nantong, Jiangsu, China; ^3^Institute of Special Environmental Medicine, Nantong University, Nantong, Jiangsu, China; ^4^Security Office, Nantong University, Nantong, Jiangsu, China

**Keywords:** self-identity, future orientation, self-continuity, parenting style, college students

## Abstract

**Introduction:**

The self-awareness of college students will have an impact on their future development, especially their mental health and employment. This study explored the relationship between self-identity and future orientation with self-continuity as a chain-mediating variable, as well as the moderating role of parenting style in the direct and indirect effects of self-identity on future orientation among college students.

**Methods:**

Data were collected from 563 college students from universities aged 18–23 years (*M* = 20.75, SD = 1.42). A self-identity scale, self-continuity questionnaire, and scale of consideration of future consequences were used to evaluate self-identity, past self-continuity, future self-continuity, and future orientation, respectively. Hayes’ PROCESS macro for SPSS was utilized to test relationships among the variables.

**Results:**

Past self-continuity and future self-continuity had an independent-mediating and chain-mediating effect on the relationship between self-identity and future orientation, respectively. Parenting style had a moderating effect in the chain-mediation model. Specifically, among those reporting democratic parenting, self-identity is a stronger positive predictor of past self-continuity, and among those reporting non-democratic parenting, past self-continuity was a stronger negative predictor of future orientation.

**Conclusion:**

Self-continuity is a critical mediating mechanism through which self-identity is associated with future orientation among college students, and self-reported parenting style serves as a moderating variable in the indirect influence of self-identity on future orientation. These findings underscore the importance of considering both individual and environmental factors in shaping the future trajectories of college students.

## 1 Introduction

The COVID-19 pandemic has caused global panic and dilemmas, even as we transition into the post-pandemic era, the profound impact of this crisis on society remains a significant concern. Higher education in China in particular, has faced unprecedented challenges marked by uncertainty and instability, including college students’ change of learning mode, prominence of emotional problems and unprecedented employment pressure. How to get rid of the dilemma is an important challenge for the growth of contemporary college students. Researches have confirmed that individuals’ future orientation level positively influences their academic performance, mitigation of negative emotions and career decision-making (Zheng et al., 2019; Mac Giollabhui et al., 2018; Zhang and Wei, 2018; Zhang et al., 2022; Bowles, 2008; Liu et al., 2017). Future orientation refers to an individual’s preferred direction of thought and behavioral concerning the future, as well as the thinking and planning process for future outcomes (Liu et al., 2010). It correlates with self-esteem positively (Wang, 2014), plays a protective role in the stress-depression relationship (Zheng et al., 2019; Mac Giollabhui et al., 2018), which in turn significantly positively predicts academic achievement (Zhang and Wei, 2018; Bowles, 2008) and alleviates problematic behaviors as well as maladjustment (Wills et al., 2001). Furthermore, study also believes that future orientation is a pivotal factor influencing individuals’ long-term career development (Ferrari et al., 2010). College students’ with a stronger future orientation level tend to encounter fewer difficulties in career decision-making processes (Liu et al., 2017).

As a part of individual socialization, future orientation is shaped by a combination of social environment and individual characteristics. Factors such as family socioeconomic status (Chen and Xu, 2020), social support (Dai, 2008) and school atmosphere (Li et al., 2017) all exert significant influence on individuals’ future orientation. Previous studies have pointed out that different parenting styles lead to different future planning and decision-making, individuals with authoritarian parenting styles have better career planning and higher career decision making self-efficacy (Zhang, 2019). In addition, several personal characteristics, including gender (Zhou, 2006; Zhang et al., 2006), age (Wang, 2011) and personality (He, 2012), have also been proven to affect college students’ future orientation. For example, He Liming reported that personality traits significantly predict college students’ degree of future orientation, with agreeableness, extraversion, emotional stability, and conscientiousness showing notable correlations with students’ future orientation of education, occupation and family (He, 2012). Furthermore, the development of an individual’s self-identity is also believed to impact future orientation. Self-identity, which refers to individuals’ subjective experience and perception about their own abilities, cognitive worlds, and life development status (Jiang et al., 2017). A stronger self-identity corresponds to a stronger future orientation (He, 2012; Huang and Zheng, 2000). Self-continuity is the meaningful connection between an individual’s past, present, and future in different periods, leading to a unity of the self (Chandler and Proulx, 2008). Self-continuity is divided into two dimensions, past self-continuity refers to the personal perception of continuity between the past and present selves, and future self-continuity refers to the perception of continuity between the present and future selves. One research has confirmed that a higher the degree of continuity and consistency between an individual’s present and future selves is associated with a stronger future orientation; this relationship also holds true for future self-continuity, whereas the relationship with past self-continuity remains less clear (Liu et al., 2018).

Although previous studies have confirmed that future orientation can be affected by individual and environmental factors, it is not clear how and why self-continuity mediates the link between self-identity and future orientation. Consequently, our ongoing study focuses on the mechanism influencing college students’ future orientation in the post-epidemic era. Specifically, we explore the relationship between college students’ self-identity and future orientation as well as the chain-mediating role of self-continuity (past self-continuity and future self-continuity). We also investigate the potential moderating role of self-reported parenting style in the direct and indirect effects of self-identity on future orientation among college students.

## 2 Methods

### 2.1 Participants and procedures

Data collection began in April 2021, this study randomly recruited college students from universities in Jiangsu Province, China, a total of 619 data were collected both online (electronic questionnaire on the Wen Juan Xing public online platform) and offline within 7 months. Before starting to fill out the questionnaires, the participants were reminded to respond to all questions in the survey based on their own feelings and perceptions. They read the instructions and information sheet explaining the research objectives and key principles (e.g., anonymity, no-harm, and confidentiality). After the participants gave their consent, they were directed to the survey questions. When there were questions left unanswered, remind information directed participants to the unanswered question, allowing them to provide their responses. Thus, the final data set did not have missing values.

The inclusion criteria for analysis were (Zheng et al., 2019) informed consent to participate and (Mac Giollabhui et al., 2018) presence of normal communication, reading, and writing abilities, that is, ability to complete the questionnaire independently. The exclusion criteria were (Zheng et al., 2019) similar answers for all items; and (Mac Giollabhui et al., 2018) response durations were shorter or longer than three standard deviations from the mean completion time. Ultimately, a total of 563 valid data were subjected to analysis.

The study involving humans was approved by Nantong University Academic Ethics Committee. The study was also conducted in accordance with the local legislation and institutional requirements. The participants provided their written informed consent to participate in this study.

### 2.2 Measures

#### 2.2.1 Self-identity scale (SIS)

The SIS was used to assess participants’ self-identity. Ochse and Plug (1986) created the SIS based on Erikson’s theory of self-psychoanalysis (Li and Lou, 2009). It includes 19 items scored on a 1–4 scale (1 for “totally not applicable” and 4 for “fully applicable”), and a high score on the scale indicates a high sense of self-identity (e.g., “I feel that my lifestyle is very suitable for me” and “When there are no acquaintances around me, I feel freer to be who I really am”). The scale has been widely used to measure college students’ self-identity and has demonstrated strong reliability and validity (Li and Lou, 2009). In this study, the internal consistency coefficient (Cronbach’s *α*) for the scale was 0.79, the questionnaire has good reliability.

#### 2.2.2 Self-continuity questionnaire

The self-continuity questionnaire was developed by Fan (2017) and used to measure participants’ perception of themselves on a continuum from the past to the present and the future. The questionnaire includes two dimensions of past self-continuity and future self-continuity and consists of seven items, all of which are scored on a 1–6 scale (1 representing “completely inconsistent” and 6 representing “completely consistent”). The higher the score, the higher the degree of consistency and continuity of self-identification from the past to the present and to the future (e.g., “Although my self-awareness is constantly changing, it remains consistent in most cases” and “I know I am still my original self”). In this study, the scale’s Cronbach’s *α* was 0.86, indicating good internal consistency.

#### 2.2.3 Scale of consideration of future consequences (CFC)

Future orientation was assessed with the scale of consideration of future consequences, which was developed by Strathman et al. (1994). In this study, the Chinese version was used and demonstrated good reliability and validity (Ji et al., 2013). The scale is unidimensional, comprising 12 items scored on a 5-point scale (1 representing “very inconsistent” and 5 representing “very consistent”). Higher scores indicate stronger future orientation (e.g., “I will consider how things will develop in the future and try to influence those things through daily actions” and “I am willing to sacrifice current happiness and happiness in exchange for future achievements”). In this study, the Cronbach’s *α* for the scale was 0.78 (with good reliability).

### 2.3 Data analyses

For cases with missing values, the average value substitution method is used for data organization. The first step of data analysis was to test correlations among variables using descriptive statistics and Pearson correlation analysis. Then, in SPSS 22.0, we used the bootstrap test in the PROCESS macro (model 6 and model 92) to verify the moderated chain-mediating effect, 95% confidence intervals were computed for regression coefficients using 5,000 re-samplings (Hayes, 2013). If the bias-corrected bootstrap (95% confidence intervals) did not include 0, it indicated a significant mediating or moderating effect at the α = 0.05 level.

## 3 Results

### 3.1 Subject demographic data

Among the valid surveyed participants, 271 were male, accounting for 48.1%, while 292 were female, comprising 51.9%. Their ages ranged from 18 to 23 years, *Mage* = 20.75 years, *SD* = 1.42. Regarding parenting styles, 59.0% of the participants (*n* = 332) reported experiencing democratic parenting style, whereas 41.0% (*n* = 231) reported experiencing non-democratic parenting style.

The demographic information is shown in Table 1.

**Table 1:** Demographic characteristics.

Demographic characteristics		*n*	Composition ratio (%)
Gender	Male	271	48.1
	Female	292	51.9
Only child or not	Yes	357	63.4
	No	206	36.6
Living area	Urban	341	60.6
	Rural	222	39.4
Family state	Core family	503	89.3
	Divorced or single-parent families	60	10.7
Self-reported parenting style	Democratic parenting style	332	59.0
	Non-democratic parenting style	231	41.0

### 3.2 Common method deviation test

In order to avoid common methodological deviations (Podsakoff et al., 2003), the Harman single factor method was used for statistical control. The results showed that there were 14 factors with a characteristic value greater than 1 after factor analysis. The first factor explained 13.203% of the variation, which was less than the critical value of 40%. It indicated that common method deviation was not a concern.

### 3.3 Correlations

Table 2 showed descriptive statistics and correlations among the study variables. Of note, self-identity significantly positively correlated with past self-continuity, future self-continuity, and future orientation. Additionally, future self-continuity significantly positively correlated with past self-continuity and future orientation.

**Table 2:** Descriptive statistics and correlations among the study variables.

Variables	*M*	*SD*	1	2	3	4
1 Self-identity	56.25	7.069	-			
2 Past self-continuity	13.05	2.658	0.385***	-		
3 Future self-continuity	17.23	3.508	0.446***	0.630***	-	
4 Future orientation	39.54	5.452	0.370***	−0.189*	0.256***	-

*p < 0.05, **p < 0.01, ***p < 0.001, same below.

### 3.4 Chain-mediating effect

Based on the correlational analysis, we first standardized each variable to reduce the interference of multicollinearity with the results, then we tested the chain-mediating effect of past self-continuity and future self-continuity on the relationship between self-identity and future orientation by using Model 6 of the PROCESS macro, controlling for demographic variables (Wu et al., 2011). Table 3 showed the results.

**Table 3:** Moderated chain-mediating effect.

Result variables	Predictors	*R*	*R^2^*	*F*	β	*t*
Past self-continuity	Self-identity	0.385	0.148	97.523***	0.385	9.875***
Future self-continuity	Self-identity	0.668	0.450	225.295***	0.239	7.018***
	Past self-continuity				0.538	15.790***
Future orientation	Self-identity	0.426	0.181	41.297***	0.351	5.318***
	Past self-continuity				−0.241	−4.834***
	Future self-continuity				0.251	4.884***

College students’ self-identity significantly positively predicted past self-continuity (*β* = 0.385, *p* < 0.001, 95% *CI =* [0.31, 0.46]) as well as future self-continuity (*β* = 0.239, *p* < 0.001, 95% *CI =* [0.17, 0.31]). And the result verified the independent mediating effect of past self-continuity and future self-continuity in the relationship between self-identity and future orientation, that was, self-identity significantly negatively predicted future orientation through past self-continuity (*β* = −0.093 (0.385*−0.241), *p* < 0.001, 95% *CI =* [−0.14, −0.05]) and significantly positively predicted future orientation through future self-continuity (*β* = 0.060 (0.239*0.251), *p* < 0.001, 95% *CI =* [0.03, 0.09]). In addition, the results also showed that self-identity could positively predict future orientation directly (*β* = 0.351, *p* < 0.001, 95% *CI =* [0.27, 0.44]) or indirectly through the chain mediation of past self-continuity and future self-continuity (*β* = 0.052 (0.385*0.538*0.251), *p* < 0.001, 95% *CI =* [0.03, 0.08]).

### 3.5 Moderated chain-mediating effect

Thus, we used Model 92 of the PROCESS macro to explore the moderating effect of parenting style on the above chain-mediating model. The results were in Table 4.

**Table 4:** Moderated chain-mediating effect.

Result variables	Predictors	*R*	*R^2^*	*F*	β	*t*
Past self-continuity	Self-identity	0.393	0.155	34.092***	0.226	2.608**
	Parenting style				0.098	1.031
	Self-identity × Parenting style				0.201	2.054*
Future self-continuity	Self-identity	0.670	0.450	90.952***	0.271	3.824***
	Past self-continuity				0.608	10.305***
	Parenting style				−0.106	−1.366
	Self-identity × Parenting style				−0.017	−0.202
	Past self-continuity × Parenting style				−0.102	−1.406
Future orientation	Self-identity	0.470	0.221	22.460***	0.465	5.318***
	Past self-continuity				−0.525	−6.052***
	Future self-continuity				0.369	4.426***
	Parenting style				−0.267	−2.890**
	Self-identity × Parenting style				−0.125	−1.229
	Past self-continuity × Parenting style				0.431	4.098***
	Future self-continuity × Parenting style				−0.182	−1.736

When including parenting style (0 for non-democratic parenting style, 1 for democratic parenting style) in each action path in the chain-mediating model, the interaction of self-identity and parenting style significantly predicted past self-continuity (*β* = 0.201, *p* < 0.05, 95% *CI* = [0.01, 0.39]), and the interaction of past self-continuity and parenting style significantly predicted future orientation (*β* = 0.431, *p* < 0.001, 95% *CI* = [0.22, 0.64]).

**Figure 1:**
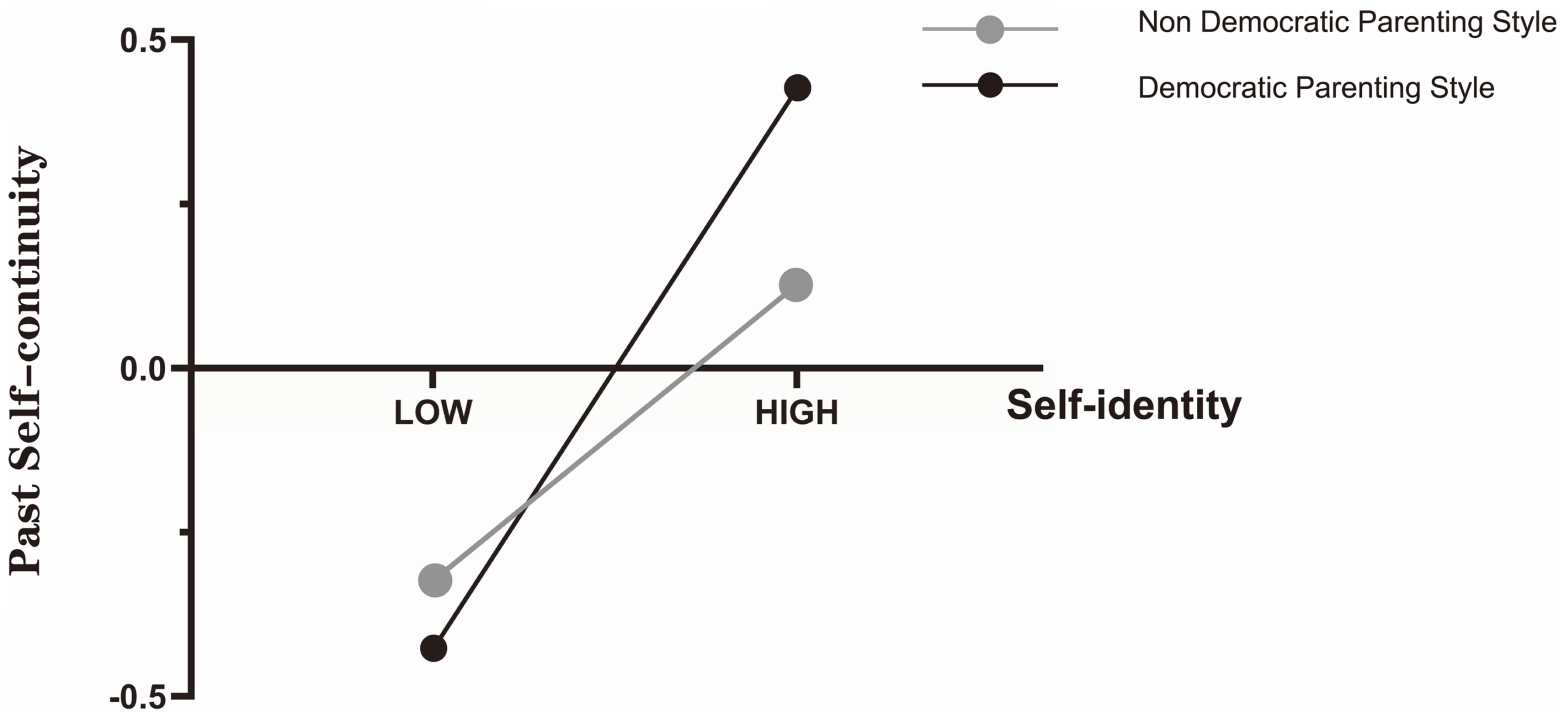
Parenting style as a moderator of the relationship between self-identity and past self-continuity.

**Figure 2:**
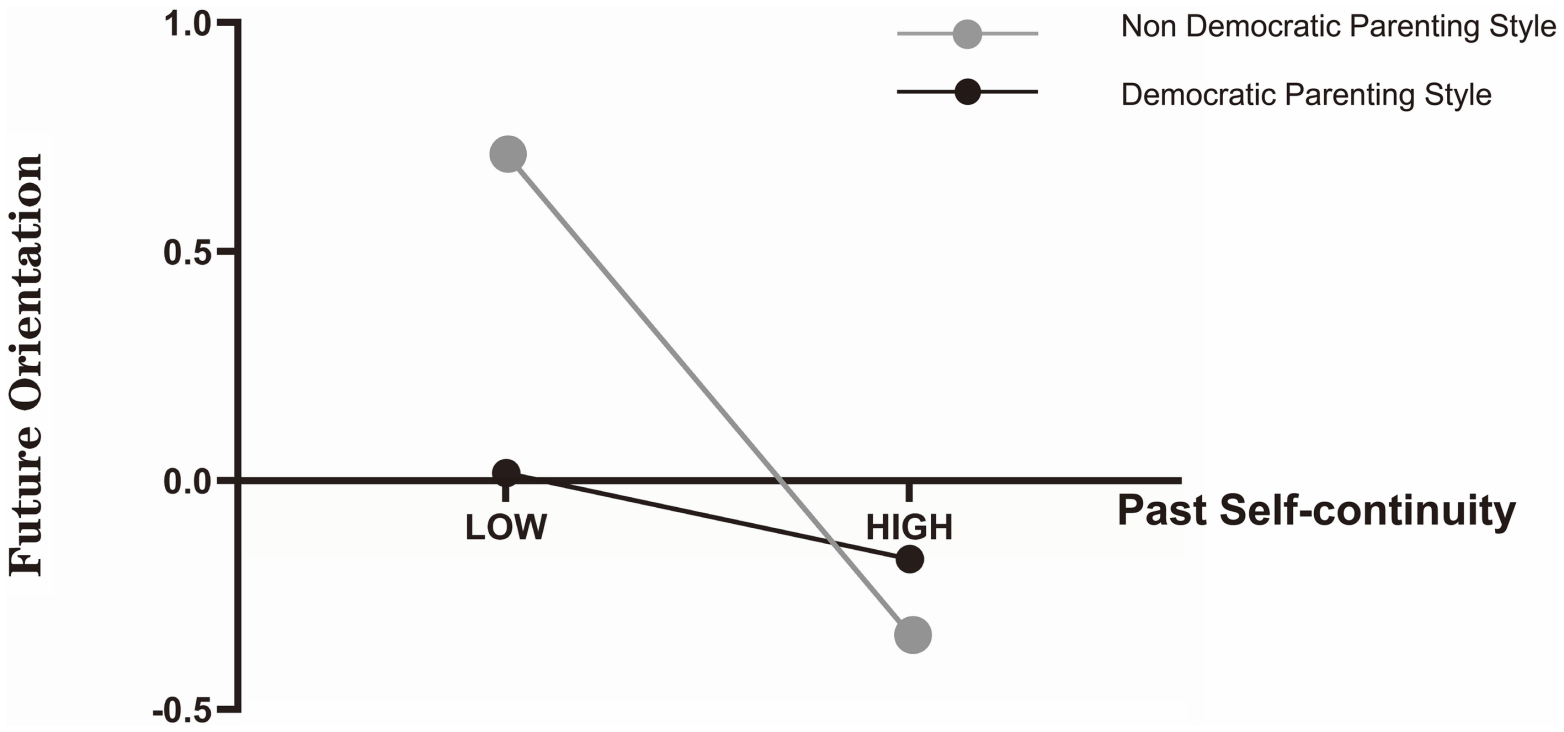
Parenting style as a moderator of the relationship between past self-continuity and future orientation.

To display the moderation effect more intuitively and clearly, a simple slope analysis was conducted and the effect of self-identity on past self-continuity for college students with democratic or non-democratic parenting was plotted (Figure 1). Self-identity is a stronger positive predictor of past self-continuity for those reporting democratic parenting (*β*_simple_ = 0.426, *p* < 0.001, 95% *CI* = [0.33, 0.52]) compared to those reporting non-democratic parenting (*β*_simple_ = 0.225, *p* < 0.01, 95% *CI* = [0.06, 0.39]). Similarly, we also performed a simple slope analysis and plotted the effect of past self-continuity on future orientation for college students with democratic or non-democratic parenting (Figure 2). The figure showed that when compared with those reporting democratic parenting (*β*_simple_ = −0.094, *p* > 0.05, 95% *CI* = [−0.21, 0.02]), past self-continuity among those reporting non-democratic parenting had a stronger negative impact on future orientation (*β*_simple_ = −0.524, *p* < 0.001, 95% *CI* = [−0.69, −0.35]).

**Figure 3:**
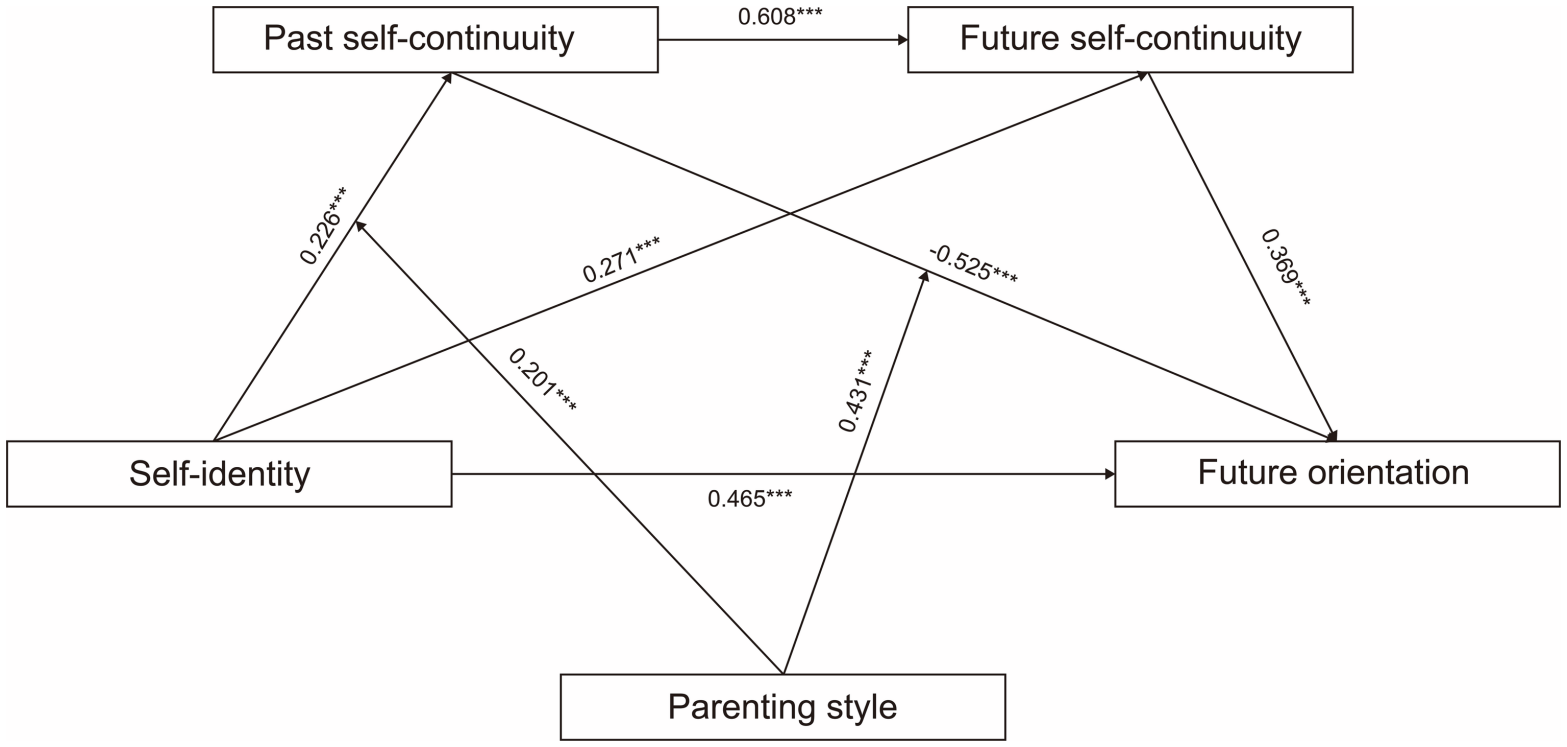
Diagram of moderated chain mediation (standardized).

Based on the above results, Figure 3 showed the moderated chain-mediating model. The hypothesized relationships in the model are supported by the results.

## 4 Discussion

This study focused on the relationship between college students’ self-identity and future orientation and tested the chain-mediating effects of past self-continuity and future self-continuity. Through the mediator of self-continuity, the causal relationship between self-identity and future orientation had been elucidated. It was a theoretical expansion of the study of the self and its relationship with future orientation. We also explored parenting style as a potential moderating variable and constructed a moderated chain-mediating model to test the hypotheses. Results not only clarified how college students’ self-identity affects their future orientation (past self-continuity and future self-continuity played mediating roles) but also revealed the influence of (non-)democratic parenting style. These insights illuminate the current psychological state of college students and propose effective strategies to promote their future orientation in the post-COVID-19 era.

### 4.1 College students’ self-identity predicts their future orientation

In the process of self-development, individuals with strong self-identity tend to have a subjectively stronger sense of consistency, continuity, and unity (Jiang et al., 2017; Liu and Liang, 2022; Li, 2013). Holding more positive attitudes toward the future has been linked to a heightened likelihood of future success (Zhang et al., 2009), as well as enhanced future career flexibility and professional confidence (Cheng et al., 2016; Fusco et al., 2018). Our study reveals that college students’ self-identity significantly positively predicts their future orientation, aligning with previous findings (Huang and Zheng, 2000; Huang, 2004). For instance, Huang and Zheng (2000) used an experimental approach to investigate the psychological structure of time and self-integration among college students in terms of breadth, direction, and relevance of time, their results confirmed that college students with stronger self-identity display a more optimistic and open attitude toward the future.

### 4.2 The chain-mediating effect of past self-continuity and future self-continuity on the relationship between self-identity and future orientation

Both past self-continuity and future self-continuity affect the individual’s current perceptions and future decision-making behaviors (Hershfield et al., 2011). Our study verified the independent mediating effect and chain-mediating effect of past self-continuity and future self-continuity in the relationship between self-identity and future orientation. Notably, the directions of these independent mediating effects diverged: Self-identity negatively predicted future orientation through past self-continuity, but it positively through future self-continuity. When college student are overly focused on their past selves, their future orientation tends to weaken, potentially reducing their willingness to embrace new challenges and making them more content with the status quo. On the contrary, college students enrich new life and learning experiences, enabling a deeper understanding of their future selves, enhancing individual plasticity and directing their cognition and behavior toward the future (i.e., future-oriented self-perceptions strengthen overall future orientation).

Our study further validated that college students’ self-identity positively predicted their future orientation through self-continuity. The stronger one’s self-identity, the greater the continuity between their past, present, and future selves, and consequently, the stronger one’s future orientation. This chain-mediating effect further emphasized that the high positive correlation between past and future self-continuity was the key to positively predicting future orientation of self-identity. The negative influence of the independent mediating effect of past self-continuity will hinder the development of future orientations among college students, while future self-continuity could promote and predict the development of more positive future orientations, as previously demonstrated in study showing its positive correlation with academic performance and employment intentions (Li, 2019; Chen, 2022).

### 4.3 The moderating effect of parenting style in the relationship between self-identity and future orientation

Family education is very important to individuals’ growth, and parenting style affects the formation of self-identity (Feng and Gao, 2022). The findings of this study revealed that the parenting style significantly moderates the relationship between self-identity and past self-continuity, and among those reporting democratic parenting, self-identity was a stronger positive predictor of past self-continuity, highlighting the pivotal developmental impact of parenting style. Studies have also confirmed that a supportive family system strengthens individuals’ future orientation, and a positive and authoritative parenting style enhances planning abilities concerning future education and careers (Zhang, 2019; Zhang, 2008). In the relationship between past self-continuity and future orientation, democratic parenting style did not play a significant predictive role, whereas among those reporting non-democratic parenting, past self-continuity emerged as stronger negative predictor of future orientation. Therefore, a nurturing parenting style may be a vital factor in cultivating health-related behaviors during personal growth, particularly during the journey of past self-continuity (Park and Walton-Moss, 2012). On the other hand, the moderating effect of parenting style was not significant in the relationship between future self-continuity and future orientation, which might be due to the gradual weakening of parents’ involvement on individuals’ thinking and planning for the future.

### 4.4 Implications and limitations

Our results have several significant implications. Firstly, this study confirms that the roles of past and future self-continuity in the relationship between self-identity and future orientation are diametrically opposed, highlighting the adverse effects of past self-continuity on future development. It also suggests the positive effect of correct awareness of present and future selves on college students’ future orientation. Secondly, it reveals the factors influencing future orientation through a moderated chain-mediating model. This model offers initial exploration into enhancing college students’ self-perceptions through interventions aimed at developing future orientation and improving future career decisions. For instance, in the process of guiding college students’ employment, by promoting their self-awareness and thinking, an internal incentive mechanism can be formed to effectively guide their future behavioral orientation. Thirdly, the study emphasizes the moderating effect of parenting style, suggesting that the impact of family education should not be ignored in longitudinal studies on self-development. By creating an open democratic family upbringing environment, college students can promote positive self-cognition and effectively guide to future behavior.

It’s important to note that the study also has some limitations. Firstly, the sample is limited to college students, and the relevance of the conclusions may not apply to other student groups, such as middle school students, primary school students, etc. To enhance representativeness, future research should incorporate samples from a broader range of educational levels and settings or explore differences and commonalities of specific faculty, in order to enrich the research results. Secondly, the data were all collected from college students’ self-report, it faced risk of common method bias. Furthermore, due to the constraint of the number of items in the current research questionnaires, parenting style was only evaluated through self-report of the subjects, without the utilization of a standard parenting scale, so there was a lack of more objective evaluation indicators. Lastly, the present research adopts a horizontal design, which may not fully reflect individuals’ developmental status. Therefore, causal relationships in these data should be interpreted cautiously. Future research may consider implementing a longitudinal design.

## 5 Conclusion

Past self-continuity and future self-continuity have independent mediating and chain-mediating effects in the relationship between self-identity and future orientation, this underscores the importance of understanding how individuals perceive their past and future selves in shaping their future orientations. Furthermore, the moderating effect of parenting style in the chain-mediating model reveals that early life experiences and family education play a pivotal role in shaping these orientations. In the complex social environment, college students are facing high growth pressure and fierce employment landscape. This research provides crucial insights for educators, counselors, and policymakers. By fully considering the developmental status and family background of college students, we can design more tailored interventions to strengthen their future orientation.

## Data Availability

The raw data supporting the conclusions of this article will be made available by the authors, without undue reservation.

## References

[ref1] Bowles T. (2008). The relationship of time orientation with perceived academic performance and preparation for assessment in adolescents. Educ. Psychol. 28, 551–565. doi: 10.1080/01443410701880134, PMID: 40101104

[ref2] Chandler M. J. Proulx T. (2008). “Personal persistence and persistent peoples: continuities in the lives of individual and whole cultural communities” in Self-continuity: Individual and collective perspectives. ed. Sani F. (New York: Psychology Press).

[ref3] Chen Y. N. (2022). Research on the relationship between college students' ingenuity, future self-continuity and entrepreneurial intention. Unpublished master’s thesis. Jiangsu, China: Yangzhou University.

[ref4] Chen J. J. Xu L. (2020). Why is it more difficult for junior high school students with low socioeconomic status to achieve academic success— the multiple intermediary role of educational values and future orientation. Educ. Sci. Res. 12, 33–38.

[ref5] Cheng C. Yang L. Chen Y. Zou H. Su Y. Fan X. (2016). Attributions, future time perspective and career maturity in nursing undergraduates: correlational study design. BMC Med. Educ. 16:26. doi: 10.1186/s12909-016-0552-1, PMID: 26810472 PMC4727268

[ref6] Dai B. B.. Factors influencing teenagers' future orientation. Unpublished master’s thesis, Capital Normal University, Beijing, China, (2008).

[ref7] Fan L. (2017). A study on the relationship between nostalgia, self-continuity and college students' adaptation. Unpublished master’s thesis. Hubei, China: Huazhong University of Science and Technology.

[ref8] Feng X. H. Gao C. Y. (2022). The influence of parental rearing styles on adolescent self-identity: the mediation of parent-child relationship. Modern Occupat Educ. 41, 76–79.

[ref9] Ferrari L. Nota L. Soresi S. (2010). Time perspective and indecision in young and older adolescents. Brit. J. Guid. Couns. 38, 61–82. doi: 10.1080/03069880903408612

[ref10] Fusco L. Sica L. S. Boiano A. Esposito S. Sestito L. A. (2018). Future orientation, resilience and vocational identity in southern Italian adolescents. Int. J. Educ. Vocat. Guidance 19, 63–83. doi: 10.1007/s10775-018-9369-2, PMID: 40130261

[ref11] Hayes A. F. (2013). Introduction to mediation, moderation, and conditional process analysis: a regression-based approach. New York, NY, USA: Guilford Press, xvii-507.

[ref12] He M. L. (2012). Research on the influencing factors and mechanism of college students' future orientation. Unpublished master’s thesis. Guangdong, China: Guangzhou University.

[ref13] Hershfield H. E. Goldstein D. G. Sharpe W. F. Fox J. Yeykelis L. Carstensen L. L.. (2011). Increasing saving behavior through age-progressed renderings of the future self. J. Mark. Res. 48, S23–S37. doi: 10.1509/jmkr.48.SPL.S23, PMID: 24634544 PMC3949005

[ref14] Huang X. T. (2004). The theory of temporal insight. Psychol. Sci. 1, 5–7. doi: 10.16719/j.cnki.1671-6981.2004.01.002

[ref15] Huang X. T. Zheng Y. (2000). Self-integration of time perspective: projective test of psychological structure. Acta Psychol. Sin. 10, 261–269. doi: 10.1007/s11769-000-0010-0, PMID: 40130261

[ref16] Ji W. B. Wang L. Mo H. Y. Liu J. Cheng Y. W. (2013). The effect of expectation on adolescent aggressive behavior: mediation effect and moderation effect. Psychol. Dev. Educ. 29, 86–93. doi: 10.16187/j.cnki.issn1001-4918.2013.01.001

[ref17] Jiang Y. Z. Ba X. L. Liu Y. Chen Z. Y. (2017). The influence of social adaptability on adolescent mobile social network use: the chain mediation of self-identity and psychological harmony. Chin J Clin Psychol. 25, 550–553. doi: 10.16128/j.cnki.1005-3611.2017.03.035

[ref18] Li L. P. (2013). A study on the status of college students' self-identity and its relationship with vocational maturity. Unpublished master’s thesis. China: Yunnan Normal University.

[ref19] Li D. W. (2019). The effect of future self-continuity on academic engagement of college students: Moderating mediating effect. Unpublished master’s thesis. Hubei, China: Huazhong University of Science and Technology.

[ref20] Li W. T. Liu X. L. Yu C. P. Zhang C. X. Ye P. Y. (2017). School atmosphere and academic achievement of junior middle school students: mediation of academic emotion and regulation of future orientation. Psychol. Dev. Educ. 33, 198–205. doi: 10.16187/j.cnki.issn1001-4918.2017.02.09

[ref21] Li Y. A. Lou W. J. (2009). The reliability and validity of self-identity scale in adolescent students. Chin. J. Health Psychol. 17, 181–182. doi: 10.13342/j.cnki.cjhp.2009.02.057

[ref22] Liu W. Z. Chen Z. D. Hu D. M. Si J. W. (2017). The relationship between college students' future time insight and career decision-making difficulties: the intermediary role of career maturity. Psychol. Res. 10, 73–78.

[ref23] Liu X. Huang X. T. Pu B. Bi C. H. (2010). Overview of future orientation research. Adv. Psychol. Sci. 18, 385–393.

[ref24] Liu Y. Liang J. L. (2022). Deaf, hearing or other—— self-identification of hearing children in deaf families. Chin. J. Spec. Educ. 3, 48–55.

[ref25] Liu Y. Z. Yang Z. Y. Wang Y. Q. Chen Y. Cai H. J. (2018). Future self-continuity and its influence on individual psychology and behavior. Adv. Psychol. Sci. 26, 2161–2169. doi: 10.3724/SP.J.1042.2018.02161

[ref26] Mac Giollabhui N. Nielsen J. Seidman S. Olino T. M. Abramson L. Y. Alloy L. B. (2018). The development of future orientation is associated with faster decline in hopelessness during adolescence. J. Youth Adolesc. 47, 2129–2142. doi: 10.1007/s10964-017-0803-4 29305672 PMC6033687

[ref27] Ochse R. Plug C. (1986). Cross-cultural investigation of the validity of Erikson’s theory of personality development. J. Pers. Soc. Psychol. 50, 1240–1252. doi: 10.1037/0022-3514.50.6.1240

[ref28] Park H. Walton-Moss B. (2012). Parenting style, parenting stress, and Children's health-related Behaviors. J. Dev. Behav. Pediatr. 33, 495–503. doi: 10.1097/DBP.0b013e318258bdb8, PMID: 22772823

[ref29] Podsakoff P. M. Mac Kenzie S. B. Lee J. Y. Podsakoff N. P. (2003). Common method biases in behavioral research: a critical review of the literature and recommended remedies. J. Appl. Psychol. 88, 879–903, PMID: 14516251 10.1037/0021-9010.88.5.879

[ref30] Strathman A. Gleicher F. Boninger D. S. Edwards C. S. (1994). The consideration of future consequences: weighing immediate and distant outcomes of behavior. J. Pers. Soc. Psychol. 66, 742–752. doi: 10.1037/0022-3514.66.4.742

[ref31] Wang S. Q. (2011). A follow-up study on the relationship between adolescents' future orientation and academic and emotional adaptation. Unpublished master’s thesis. China: Shandong Normal University.

[ref32] Wang Q. (2014). The relationship between college students' future orientation and self-esteem. J. Neijiang Normal Univ. 4, 43–47. doi: 10.3969/j.issn.1671-1785.2014.04.011

[ref33] Wills T. A. Sandy J. M. Yaeger A. M. Cleary S. D. Shinar O. (2001). Coping dimensions, life stress, and adolescent substance use: a latent growth analysis. J. Abnorm. Psychol. 110, 309–323. doi: 10.1037/0021-843X.110.2.309 11358025

[ref34] Wu Y. Wen Z. L. Hou J. T. Marsh H. W. (2011). Standardized estimation of latent variable interaction model without mean structure. Acta Psychol. Sin. 43, 1219–1228. doi: 10.3724/SP.J.1041.2011.01219

[ref35] Zhang L. L. (2008). The relationship between the development of adolescents' future orientation and family and peer factors. Unpublished master’s thesis. China: Shandong Normal University.

[ref36] Zhang B. Y. (2019). The relationship between parental rearing style, career decision-making self-efficacy and future career planning of senior high school students. Unpublished master’s thesis. Shandong, China: University of Jinan.

[ref37] Zhang Q. Miao L. He L. Wang H. (2022). The relationship between self-concept and negative emotion: a moderated mediation model. Int. J. Environ. Res. Public Health 19:10377. doi: 10.3390/ijerph191610377, PMID: 36012021 PMC9407814

[ref38] Zhang F. Wei Y. G. (2018). The relationship between future orientation, academic burn out and happiness of senior high school students. Mental Health Educ. Prim. Sec. Sch. 381, 6–8. doi: 10.3969/j.issn.1671-2684.2018.34.002

[ref39] Zhang W. X. Xu F. Z. Zhang L. L. Wang S. Q. Yu F. J. Gao T. (2009). College students' planning and attitudes towards personal future and their relationship with depression. Psychol. Sci. 32, 824–827. doi: 10.16719/j.cnki.1671-6981.2009.04.063

[ref40] Zhang L. L. Zhang W. X. Ji L. Q. (2006). The measurement analysis of Chinese version of adolescent future orientation questionnaire. Psychol. Dev. Educ. 1, 103–108. doi: 10.3969/j.issn.1001-4918.2006.01.020

[ref41] Zheng L. Lippke S. Chen Y. Li D. Gan Y. (2019). Future orientation buffers depression in daily and specific stress. Psy Ch Journal. 8, 342–352. doi: 10.1002/pchj.283, PMID: 30945435

[ref42] Zhou X. Q. (2006). A comparative study on the future orientation of senior high school students and secondary vocational students. Unpublished master’s thesis. China: Shandong Normal University.

